# Protective effect of cinnamon extract against cobalt-induced multiple organ damage in rats

**DOI:** 10.3389/fphar.2024.1384181

**Published:** 2024-05-09

**Authors:** Bahar Isik, Bahadir Suleyman, Renad Mammadov, Seval Bulut, Bulent Yavuzer, Durdu Altuner, Taha Abdulkadir Coban, Halis Suleyman

**Affiliations:** ^1^ Department of Emergency Medicine, Faculty of Medicine, Erzincan Binali Yildirim University, Erzincan, Türkiye; ^2^ Department of Pharmacology, Faculty of Medicine, Erzincan Binali Yildirim University, Erzincan, Türkiye; ^3^ Department of Medical Biochemistry, Faculty of Medicine, Erzincan Binali Yildirim University, Erzincan, Türkiye

**Keywords:** cinnamon, cobalt, heart, kidney, liver, oxidative damage

## Abstract

**Background:**

The role of oxidative stress and inflammation in cobalt (Co) toxicity has been the focus of previous studies. Cinnamon and its main components have been reported to have protective effects in various tissues with antioxidant and anti-inflammatory effects.

**Aims:**

In this study, the protective effect of cinnamon extract (CE) against possible Co-induced heart, kidney, and liver damage in rats was investigated biochemically.

**Methods:**

Eighteen *albino Wistar*-type male rats were categorized into three groups (*n* = 6 per group): control (CG), CoCL_2_-administered (CoCL_2_), and CE + CoCL_2_-administered (CE + Co) groups. The CE + CoCL_2_ group was administered CE (100 mg/kg), and the CoCL_2_ and CG groups were administered distilled water orally by gavage. One hour after the administration, Co (150 mg/kg) was administered orally to the CE + CoCL_2_ and CoCL_2_ groups. This procedure was repeated once daily for 7 days. Then, biochemical markers were studied in the excised heart, kidney, and liver tissues.

**Results:**

CoCL_2_ increased oxidants and proinflammatory cytokines and decreased antioxidants in heart, kidney, and liver tissues. Heart, kidney, and liver tissue were affected by Co damage. CE treatment suppressed the CoCL_2_-induced increase in oxidants and proinflammatory cytokines and decrease in antioxidants in heart, kidney, and liver tissues. CE treatment has been shown to attenuate cardiac damage by reducing serum troponin I (TpI) and creatine kinase-MB (CK-MB), renal damage by reducing creatinine and blood urea nitrogen (BUN), and liver damage by reducing alanine aminotransferase (ALT) and aspartate aminotransferase (AST).

**Conclusion:**

Co induced the production of oxidants and proinflammatory parameters and antioxidant depletion in heart, kidney, and liver tissues of rats. Our experimental results show that CE protects heart, kidney, and liver tissues against oxidative and inflammatory changes induced by CoCLl_2_.

## 1 Introduction

Cobalt (Co) is a hard, silver-gray-colored trace element that is commonly found in the natural environment (Leyssens et al., 2017). Trace elements participate in the structure of enzymes and cofactors, and therefore, their presence in small amounts in the body is important for the fulfillment of physiological functions. They are involved in the prevention of nutritional deficiencies, regulation of immune responses, antioxidant defense systems, and gene activities, as well as in the prevention of the development of chronic diseases (Strachan, 2010; Leyssens et al., 2017). Co is also essential for health as a structural component of vitamin B12. However, overexposure to Co components has been shown to cause several adverse effects (Faroon and Keith, 2004). Due to its widespread presence in the natural environment, humans are frequently exposed to various Co compounds through inhaled air, consumption of cobalt-containing foods and drinking water, and occupational exposure (in the metalworking industry) (Leyssens et al., 2017). Systemic toxic reactions can occur when Co ions from different routes enter the bloodstream and lymphatic circulation and spread to different organs (Harbak, and Bennekou, 2012).

Molecular mechanisms of the toxic effect of free Co^2+^ ions include increased reactive oxygen species (ROS) production and lipid peroxidation (LPO) (Paustenbach et al., 2013; Leyssens et al., 2017). In addition, it has been reported that the cobalt (II)-chloride (CoCL_2_) component causes a decrease in antioxidants and an increase in nuclear factor kappa-B (NF-κB), a proinflammatory cytokine, in heart and kidney tissues (Oyagbemi et al., 2020). Co can also induce oxidative damage in the liver by increasing oxidant production and decreasing the antioxidant capacity (Khalil et al., 2020). The cause of this Co-related toxicity is thought to be its bioaccumulation in the heart, kidney, liver, and pancreas (Harbak, and Bennekou, 2012). Information from the literature suggests that the toxic effect of Co on organs and tissues is due to an increase in oxidants and proinflammatory cytokines. These data suggest that antioxidant and anti-inflammatory drugs may be useful in reducing or completely eliminating Co-induced toxicity.

This study investigated the effects of cinnamon, the dried bark of *Cinnamomum* (Lauraceae), which has many effects, such as antioxidant, anti-inflammatory, cardioprotective, and lowering serum cholesterol levels, on the potential toxicity of Co in the heart, kidney, and liver (Yanakiev, 2020; Zheng et al., 2022). Cinnamon bark extract has been used in ischemia reperfusion injury. It has been reported that it protects cardiac tissues from oxidative damage, and this effect increases as the dose increases (Sedighi et al., 2018). Cinnamon aqueous extract has been reported to suppress neuro-inflammation via the inhibition of proinflammatory cytokines (Ho, Chang, and Chang, 2013). Cinnamon and its main constituents have been shown to protect the heart, kidney, liver, blood, brain, spleen, and reproductive system from damage by chemical toxins via their antioxidant, radical scavenging, LPO-suppressing, and anti-inflammatory effects (Dorri, Hashemitabar, and Hosseinzadeh, 2018). In the literature, there is no information on the prophylactic effect of cinnamon extract against Co-induced oxidative and inflammatory damage to the heart, kidney, and liver. Therefore, this study was designed to evaluate the prophylactic effect of cinnamon extract (CE) on possible oxidative and inflammatory heart, kidney, and liver damage induced by Co in rats.

## 2 Materials and methods

### 2.1 Animals

Eighteen male *albino Wistar* rats (250–270 g, 6–7 weeks old) were purchased from the Experimental Animal Research and Application Center of Erzincan Binali Yildirim University. The animals were brought to the laboratory environment where the experiment would be carried out in order to adapt to the environment 1 week in advance. The animals were housed at a temperature of 22°C ± 2°C, 12 h in darkness and 12 h in light, and humidity levels were 30%–70%. The animals were fed *ad libitum* with standard pellet chow (experimental animal feed; Bayramoglu AS, Erzurum, Türkiye) and tap water.

### 2.2 Chemicals

CE was purchased from Solgar (Leonia, United States). Each capsule contains 300 mg cinnamon extract (4:1) (bark) + 200 mg cinnamon powder (bark). Ketamine was purchased (500 mg/10 cc vial) from Pfizer Ilaçları Ltd. Sti (Istanbul, Türkiye), and Co (CoCL_2_ X 6 H_2_O; 5 g in poly bottle), from Merck (Darmstadt, Germany).

### 2.3 Experimental groups

The rats were categorized into three groups (*n* = 6/each group): control (CG), CoCL_2_ alone (CoCL_2_), and CE and CoCL_2_ (CE + CoCL_2_).

### 2.4 Experimental procedure

The CE capsule content was mixed with 20 cc distilled water, and a solution of 25 mg/mL was prepared. CE at a dose of 100 mg/kg (1 cc per rat) of the prepared solution was administered orally to the CE + CoCL_2_ group by gavage (Sedighi et al., 2018). In the CoCL_2_ and CG groups, distilled water was administered in the same way. For CoCL_2_ administration, 1 g CoCL_2_ was dissolved in 26 cc distilled water, and the solution was prepared as 38 mg/cc. CoCL_2_ at a dose of 150 mg/kg (1 cc per rat) was administered orally by gavage to the CE + CoCL_2_ and CoCL_2_ groups 1 h after CE and distilled water administration (Akinrinde and Adebiyi, 2019). This treatment protocol continued for 7 days, once a day. On the eighth day, following 12 h of fasting, all rats were euthanized with a high dose of ketamine (120 mg/kg), and their hearts, kidneys, and livers were removed. Malondialdehyde (MDA), total glutathione (tGSH), superoxide dismutase (SOD), catalase (CAT), NF-κB, tumor necrosis factor-alpha (TNF-α), interleukin-1β (IL-1β), and interleukin-6 (IL-6) levels were measured in the tissues of these organs. Troponin I (TpI), creatine kinase-MB (CK-MB), creatinine, blood urea nitrogen (BUN), alanine aminotransferase (ALT), and aspartate aminotransferase (AST) were measured in blood samples obtained from tail veins before euthanasia.

### 2.5 Biochemical analysis

#### 2.5.1 Preparation of serum for analysis

Anticoagulant-free tubes were used for blood samples taken from the rats. After clotting, the serum was separated by centrifugation at 15,000 rpm for 15 min (+4°C) and stored at −80 °C until analysis.

#### 2.5.2 Preparation of tissues for analysis

At this stage, 0.2 g of each tissue sample was weighed for biochemical analysis. Then, the samples were washed with physiological saline, and the tissues were ground to powder by means of grinding in a liquid nitrogen environment. They were then transferred to an ice-cold phosphate buffer solution. Homogenates were centrifuged at +4°C at 5,000 rpm for 20 min, and then, the clear filtrate was extracted for analysis.

#### 2.5.3 MDA, tGSH, SOD, and CAT analyses in rat tissues

Enzyme-linked immunosorbent assay (ELISA) rat kits were used for MDA, tGSH, and SOD analyses. The analysis procedure was determined according to the kits’ manuals (MDA catalog number: 10009055; GSH catalog number: 703002; SOD catalog number: 706002; Cayman Chemical Company). Calculations were performed using standard graphs. CAT determination was carried out by measuring the dissociation of H_2_O_2_ in the presence of catalase at 240 nm, according to the method suggested by Góth (1991).

#### 2.5.4 NF-κB, TNF-α, IL-1β, and IL-6 analyses in rat tissues

NF-κB levels were determined using rat ELISA kits purchased from SunRed (Shanghai, China), and TNF-α, IL-1β, and IL-6 levels were determined using rat ELISA kits purchased from Eastbiopharm (Hangzhou, China). Calculations were performed using standard graphs.

#### 2.5.5 TpI analysis in serum

Serum TpI levels were measured by enzyme-linked fluorescence assay (ELFA) using the VIDAS Ultra Kit (Marcy- I′ Etoile, France). The analysis was performed using the VIDAS instrument in accordance with the kit instructions.

#### 2.5.6 CK-MB analysis in serum

Serum creatine kinase-MB levels were measured in the Roche/Hitachi Cobas c 701 system. Using the ready-to-use test reagents, all steps of the test were performed using the immune-UV assay.

#### 2.5.7 Creatinine analysis in serum

Serum creatinine levels were measured spectrophotometrically on a Cobas 8000 autoanalyzer (Roche Diagnostics, Mannheim, Baden-Wurttemberg, Germany). This test is based on the Jaffe principle (Moore and Sharer, 2017).

#### 2.5.8 BUN analysis in serum

Serum urea levels were measured spectrophotometrically using a Cobas 8000 autoanalyzer (Roche Diagnostics, Mannheim, Baden-Wurttemberg, Germany). BUN was calculated with the formula BUN = urea × 0.48.

#### 2.5.9 ALT and AST analyses in serum

Using a Cobas 8000 autoanalyzer (Roche Diagnostics, Mannheim, Baden-Wurttemberg, Germany) with rat kits (Roche Diagnostics), serum ALT and AST activities were measured spectrophotometrically.

### 2.6 Statistical analysis

IBM SPSS Statistics for Windows software (IBM Corp, 2013 release, Armonk NY, United States) was used for statistical analysis. Biochemical results were expressed as mean ± standard error (X ± SEM). Normality of distribution was assessed using the Shapiro–Wilk test. Since all biochemical data were found to be normally distributed, one-way ANOVA was used for analysis. Homogeneity of variances was evaluated by Levene’s test. If the assumption was met, Tukey’s honestly significant difference test was applied; otherwise, the Games–Howell *post hoc* test was used. Statistical significance was defined as *p* < 0.05.

## 3 Results

### 3.1 Biochemical findings

#### 3.1.1 Oxidant and antioxidant analysis results of the heart, renal, and liver tissues

**FIGURE 1:**
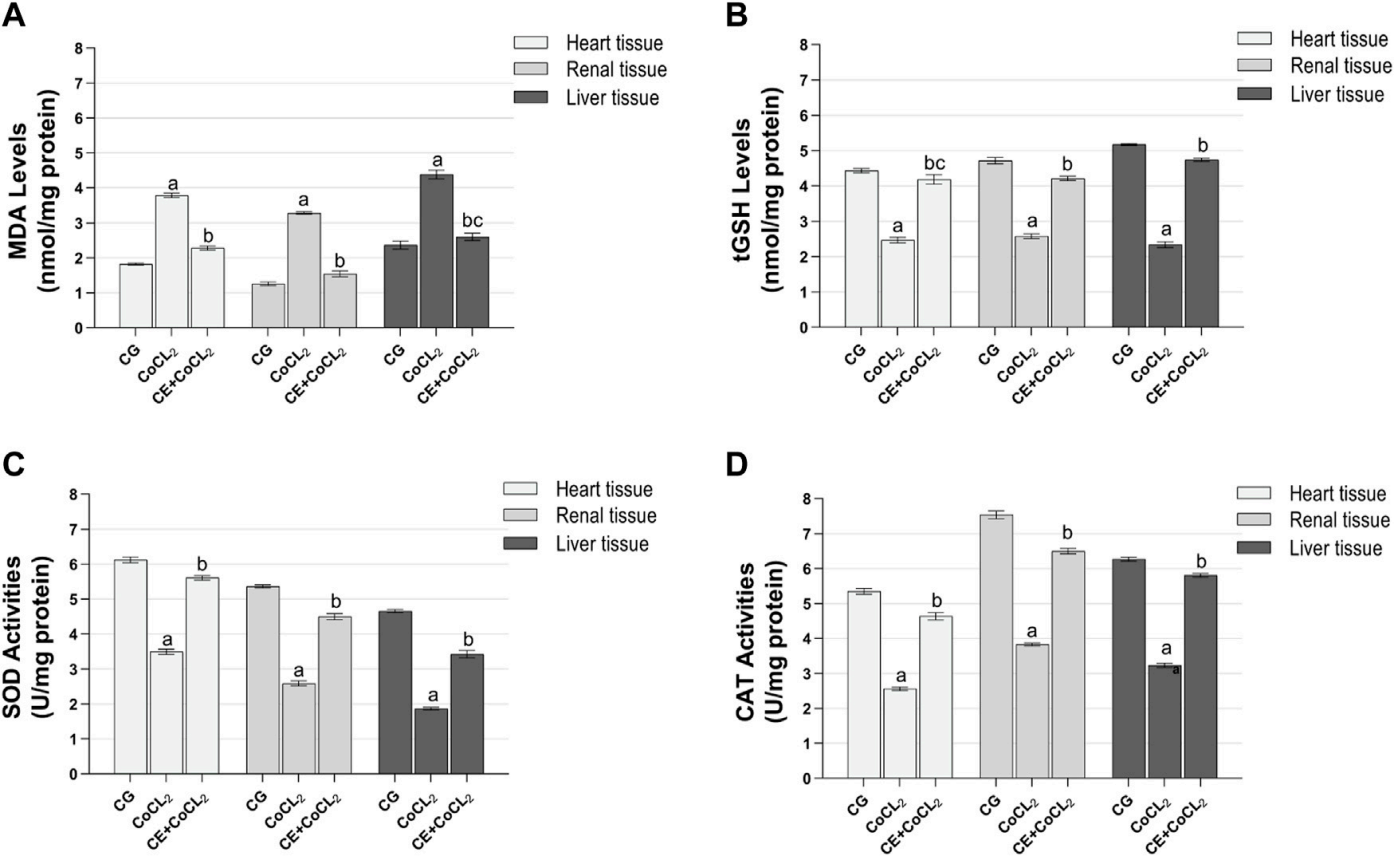
**(A–D)** Oxidative stress in heart, renal, and liver tissues of experimental groups. Bars are mean ± SEM (standard error), *n* = 6 per group. a: *p* < 0.001 vs. CG; b: *p* < 0.001 vs. CoCL_2_; c: *p* > 0.05 vs. CG; CG: control group; CoCL_2_: CoCL_2_-alone group; CE + CoCL_2_: cinnamon extract + CoCL_2_ group.

As shown in Figures 1A–D, MDA levels in the heart, renal, and liver tissues of the CoCL_2_ group were higher, whereas tGSH, SOD, and CAT levels were lower than those in the control group (*p* < 0.001). CE treatment reversed the increase in MDA levels and the decrease in tGSH, SOD, and CAT levels in the CoCL_2_ group (*p* < 0.001). No difference in MDA levels was found between the liver tissues of the CE + CoCL2 and control groups (*p* = 0.352). In terms of tGSH levels in heart tissues, control and CE + CoCL_2_ groups were similar (*p* = 0.284) (Table 1).

**TABLE 1:** Effect of cinnamon on oxidant and antioxidant levels in heart, renal, and liver tissues of cobalt-treated rats.

Biochemical variable		Mean ± SEM			*p*-values		
		CG (*n* = 6)	CoCL_2_ (*n* = 6)	CE + CoCL_2_ (*n* = 6)	CG vs. CoCL_2_	CG vs. CE + CoCL_2_	CoCL_2_ vs. CE + CoCL_2_
MDA	Heart tissue^a^	1.82 ± 0.03	3.79 ± 0.06^*^	2.28 ± 0.06^**^	<0.001	<0.001	<0.001
	Renal tissue^b^	1.26 ± 0.05	3.29 ± 0.03^*^	1.54 ± 0.08^**^	<0.001	0.043	<0.001
	Liver tissue^a^	2.37 ± 0.11	4.38 ± 0.12^*^	2.60 ± 0.11^**,***^	<0.001	0.352	<0.001
tGSH	Heart tissue^b^	4.43 ± 0.06	2.47 ± 0.08^*^	4.18 ± 0.14^**,***^	<0.001	0.284	<0.001
	Renal tissue^a^	4.72 ± 0.09	2.58 ± 0.07^*^	4.22 ± 0.06^**^	<0.001	<0.001	<0.001
	Liver tissue^a^	5.17 ± 0.03	2.34 ± 0.08^*^	4.74 ± 0.04^**^	<0.001	<0.001	<0.001
SOD	Heart tissue^a^	6.12 ± 0.08	3.49 ± 0.07^*^	5.61 ± 0.06^**^	<0.001	<0.001	<0.001
	Renal tissue^a^	5.36 ± 0.05	2.59 ± 0.07^*^	4.50 ± 0.09^**^	<0.001	<0.001	<0.001
	Liver tissue^b^	4.66 ± 0.05	1.86 ± 0.04^*^	3.43 ± 0.11^**^	<0.001	<0.001	<0.001
CAT	Heart tissue^a^	5.35 ± 0.08	2.56 ± 0.05^*^	4.64 ± 0.10^**^	<0.001	<0.001	<0.001
	Renal tissue^a^	7.54 ± 0.11	3.83 ± 0.04^*^	6.50 ± 0.08^**^	<0.001	<0.001	<0.001
	Liver tissue^a^	6.27 ± 0.05	3.23 ± 0.06^*^	5.81 ± 0.05^**^	<0.001	<0.001	<0.001

*p<0.001 vs. CG; **p < 0.001 vs. CoCL_2_; ***p > 0.05 vs. CG; SEM, standard error of the mean; n, number of animals; CG, control group; CoCL_2_, CoCL_2_-alone group; CE + CoCL_2_, cinnamon extract + CoCL_2_ group. Statistical analysis was performed by one-way ANOVA. Tukey HSD (a) or Games Howell (b) tests were used as *post hoc*. *p* < 0.05 was considered significant.

#### 3.1.2 Proinflammatory cytokine analysis results of the heart, renal, and liver tissues

**FIGURE 2:**
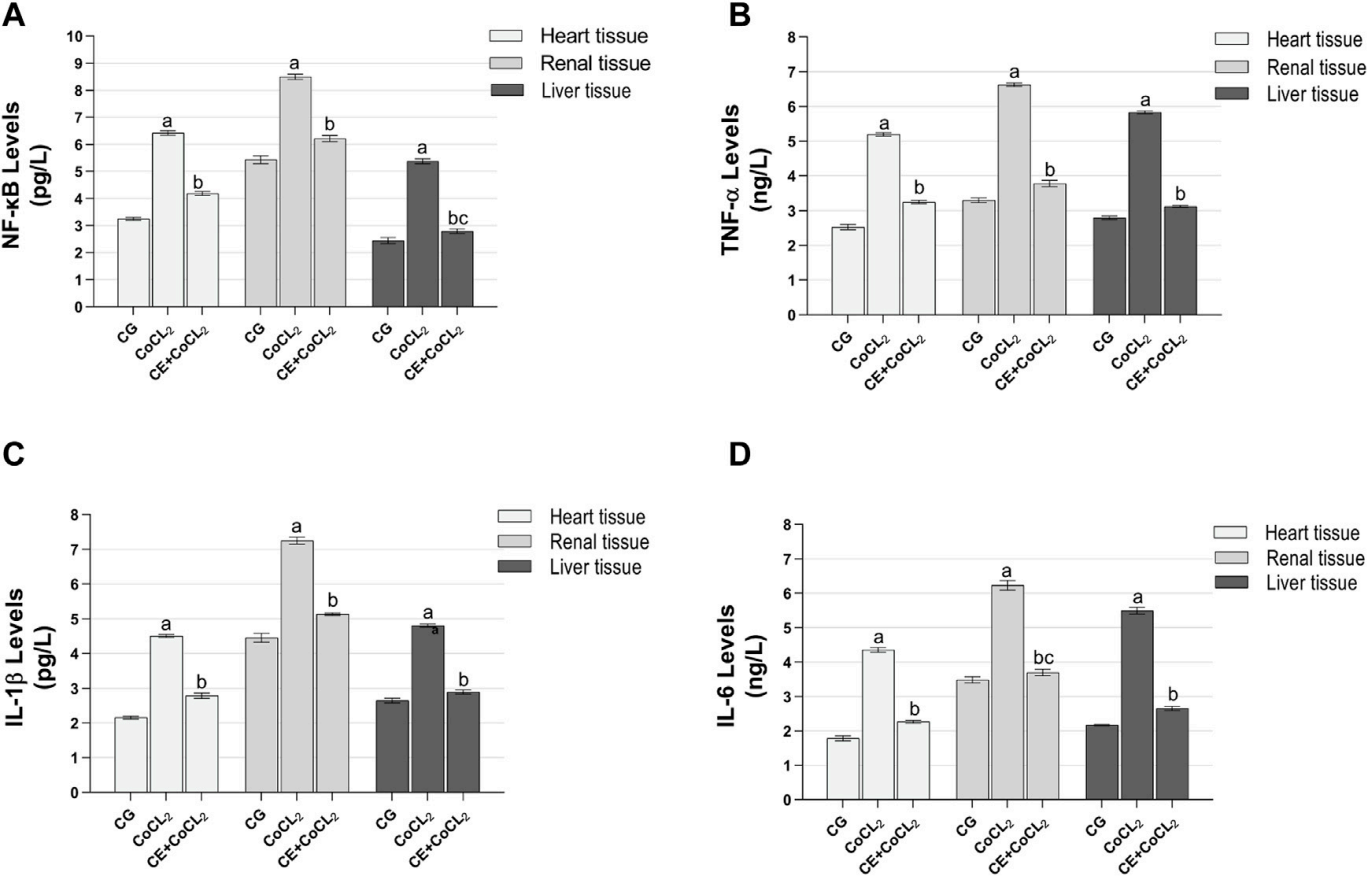
**(A–D)** Proinflammatory cytokine expressions in heart, renal, and liver tissues of experimental groups. Bars are mean ± SEM (standard error), *n* = 6 per group. a: *p* < 0.001 vs. CG; b: *p* < 0.001 vs. CoCL_2_; c: *p* > 0.05 vs. CG; CG: control group; CoCL_2_: CoCL_2_-alone group; CE + CoCL_2_: cinnamon extract + CoCL_2_ group.

NF-κB, TNF-α, IL-1β, and IL-6 levels in the heart, renal, and liver tissues of the CoCL_2_ group were higher than those of the control rats (*p* < 0.001). CE significantly inhibited the Co treatment-induced increase in NF-κB, TNF-α, IL-1β, and IL-6 levels (*p* < 0.001). NF-κB levels in CE + CoCL_2_ and control rat livers were found to be close to each other (*p* = 0.063). In addition, in terms of IL-6 levels, the data on CE + CoCL2 and CG groups were similar in kidney tissues (*p* = 0.386) (Figures 2A–D; Table 2).

**TABLE 2:** Effect of cinnamon on proinflammatory cytokine levels in heart, renal, and liver tissues of cobalt-treated rats.

Biochemical variable		Mean ± SEM			*p*-values		
		CG (*n* = 6)	CoCL_2_ (*n* = 6)	CE + CoCL_2_ (*n* = 6)	CG vs. CoCL_2_	CG vs. CE + CoCL_2_	CoCL_2_ vs. CE + CoCL_2_
NF-κB	Heart tissue^a^	3.25 ± 0.05	6.42 ± 0.09^*^	4.18 ± 0.07^**^	<0.001	<0.001	<0.001
	Renal tissue^a^	5.43 ± 0.14	8.50 ± 0.09^*^	6.22 ± 0.12^**^	<0.001	0.001	<0.001
	Liver tissue^a^	2.45 ± 0.11	5.38 ± 0.09^*^	2.79 ± 0.09^**,***^	<0.001	0.063	<0.001
TNF-α	Heart tissue^a^	2.53 ± 0.07	5.20 ± 0.05^*^	3.25 ± 0.05^**^	<0.001	<0.001	<0.001
	Renal tissue^a^	3.30 ± 0.07	6.63 ± 0.05^*^	3.78 ± 0.09^**^	<0.001	0.001	<0.001
	Liver tissue^a^	2.79 ± 0.05	5.83 ± 0.04^*^	3.12 ± 0.03^**^	<0.001	<0.001	<0.001
IL-1β	Heart tissue^a^	2.16 ± 0.04	4.51 ± 0.05^*^	2.79 ± 0.08^**^	<0.001	<0.001	<0.001
	Renal tissue^b^	4.46 ± 0.12	7.25 ± 0.10^*^	5.13 ± 0.03^**^	<0.001	0.005	<0.001
	Liver tissue^a^	2.65 ± 0.07	4.81 ± 0.05^*^	2.89 ± 0.06^**^	<0.001	0.025	<0.001
IL-6	Heart tissue^a^	1.78 ± 0.07	4.36 ± 0.07^*^	2.27 ± 0.04^**^	<0.001	<0.001	<0.001
	Renal tissue^a^	3.49 ± 0.09	6.23 ± 0.14^*^	3.70 ± 0.09^**,***^	<0.001	0.386	<0.001
	Liver tissue^b^	2.17 ± 0.02	5.50 ± 0.10^*^	2.65 ± 0.06^**^	<0.001	0.001	<0.001

*p<0.001 vs. CG; **p < 0.001 vs. CoCL_2_; ***p > 0.05 vs. CG; SEM, standard error of the mean; n, number of animals; CG: control group; CoCL_2_: CoCL_2_ alone group; CE + CoCL_2_: cinnamon extract + CoCL_2_ group. Statistical analysis was performed by one-way ANOVA. Tukey HSD (a) or Games–Howell (b) tests were used as *post hoc*. *p* < 0.05 was considered significant.

#### 3.1.3 TpI, CK-MB, creatinine, BUN, ALT, and AST analysis results of the serum

**FIGURE 3:**
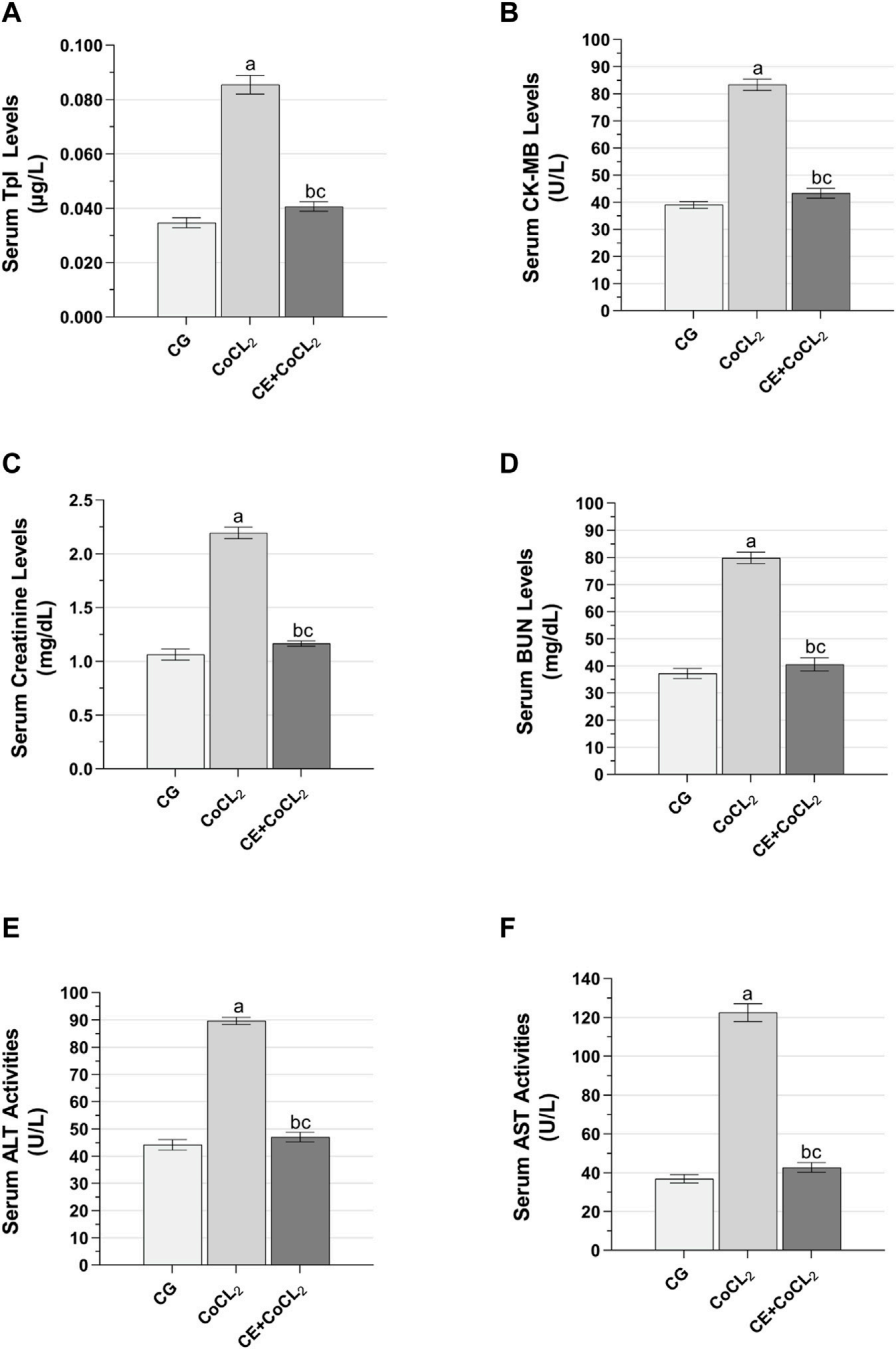
**(A–F)** TpI, CK-MB, creatinine, BUN, ALT, and AST levels in the blood serum of experimental groups. Bars are mean ± SEM (standard error), *n* = 6 per group. a: *p* < 0.001 vs. CG; b: *p* < 0.001 vs. CoCL_2_; c: *p* > 0.05 vs. CG; CG: control group; CoCL_2_: CoCL_2_-alone group; CE + CoCL_2_: cinnamon extract + CoCL_2_ group. CG: control group; CoCL_2_: CoCL_2_-alone group; CE + CoCL_2_: cinnamon extract + CoCL_2_ group.

As presented in Figures 3A, B, serum TpI and CK-MB levels of rats given Co only were higher than those of control rats (*p* < 0.001). CE treatment significantly suppressed this Co-induced increase (*p* < 0.001). No difference was found between the CoCL_2_ and CG groups in terms of serum TpI and CK-MB levels (*p* > 0.05) (Table 3).

**TABLE 3:** Effect of cinnamon on serum TpI, CK-MB, creatinine, BUN, ALT, and AST levels of cobalt-treated rats.

Biochemical variable	Mean ± SEM			*p*-values		
	CG (*n* = 6)	CoCL_2_ (*n* = 6)	CE + CoCL_2_ (*n* = 6)	CG vs. CoCL_2_	CG vs. CE + CoCL_2_	CoCL_2_ vs. CE + CoCL_2_
TpI^a^	0.035 ± 0.002	0.086 ± 0.003^*^	0.041 ± 0.002^**,***^	<0.001	0.228	<0.001
CK-MB^a^	39.00 ± 1.29	83.33 ± 2.09^*^	43.33 ± 1.82^**,***^	<0.001	0.225	<0.001
Creatinine^a^	1.06 ± 0.05	2.20 ± 0.05^*^	1.17 ± 0.03^**,***^	<0.001	0.286	<0.001
BUN^a^	37.17 ± 1.89	79.83 ± 2.09^*^	40.50 ± 2.45^**,***^	<0.001	0.532	<0.001
ALT^a^	44.17 ± 1.92	89.67 ± 1.31^*^	47.00 ± 1.77^**,***^	<0.001	0.478	<0.001
AST^a^	36.83 ± 2.18	122.50 ± 4.60^*^	42.67 ± 2.50^**,***^	<0.001	0.438	<0.001

*p<0.001 vs. CG; **p < 0.001 vs. CoCL_2_; ***p > 0.05 vs. CG; SEM, standard error of the mean; n, number of animals; CG, control group; CoCL_2_, CoCL_2_-alone group; CE + CoCL_2_: cinnamon extract + CoCL_2_ group. Statistical analysis was performed by one-way ANOVA. Tukey HSD (a) or Games–Howell (b) tests were used as *post hoc*. *p* < 0.05 was considered significant.

As evident in Figures 3C, D, the serum creatinine and BUN levels of rats receiving only Co were higher than those of control rats (*p* < 0.001). CE treatment significantly prevented this Co-induced increase in serum creatinine and BUN levels (*p* < 0.001). Data obtained from the CE + CoCL_2_ and control groups were similar in terms of serum creatinine and BUN levels (*p* > 0.05) (Table 3).

As illustrated in Figures 3E, F, ALT and AST activities in the serum of the CoCL_2_ group were higher than those in the CG group (*p* < 0.001). CE treatment suppressed the increase in ALT and AST activities (*p* < 0.001). No significant difference was noted between the CE + CoCL_2_ and CG groups in the serum ALT and AST activities (*p* > 0.05) (Table 3).

## 4 Discussion

In this study, the protective effect of CE against possible oxidative and inflammatory heart, kidney, and liver damage induced by CoCL_2_ in rats was investigated biochemically. Although Co plays a biologically essential role as the metal component of vitamin B12, overexposure is known to cause various adverse effects (Leyssens et al., 2017). Increased ROS production is involved in the pathogenesis of Co-induced toxic effects (Paustenbach et al., 2013). Superoxide anion (O_2_-¯), hydroxyl radicals (-OH), and hydrogen peroxide (H_2_O_2_) are the most studied types of ROS today (Suleyman and Ozcicek, 2019). Biochemical results of the study showed that oxidant MDA levels were higher in the heart tissues of CoCL_2_-treated animals compared with the control group, whereas antioxidant tGSH, SOD, and CAT levels were lower. MDA is one of the toxic oxidant metabolites formed as a result of peroxidation of polyunsaturated fatty acids in the cell membrane by ROS (Suleyman and Ozcicek, 2019). MDA can cause cell membrane damage and dysfunction, leading to further destruction (Suleyman and Ozcicek, 2019). Oyagbemi et al. (2020) reported that oral CoCL_2_ administration to *albino Wistar*-type rats for 8 days led to an increase in MDA levels in the heart tissue.

In this study as well, we used CoCl_2_, and tGSH levels were significantly decreased in the heart tissues of the treated rats compared with the control group. tGSH is an endogenous non-enzymatic antioxidant (Demirci-Cekic et al., 2022) and is a tripeptide structurally composed of glutaminic acid, cysteine, and glycine (Kwiecien et al., 2014). GSH is the most important reducing agent that maintains the cellular redox state by scavenging excess ROS (Tian et al., 2021; Hwang et al., 2023). In a recent study, Li et al. (2021) reported that CoCL_2_ treatment of rat embryonic cardiomyocyte cell cultures led to a decrease in tGSH levels. These findings are consistent with the result of our experiments.

In the context of the present study, CoCL_2_ also significantly decreased SOD and CAT activities in the heart tissue. As known, SOD and CAT are enzymatic antioxidants (Demirci-Cekic et al., 2022). SOD converts O_2_^−^ to H_2_O_2_, which is hydrolyzed by CAT to harmless molecules (Edzeamey et al., 2024). Wang et al. (2016) reported that CoCL_2_ treatment of embryonic rat heart cell cultures for 24 h caused a decrease in antioxidant SOD and CAT activities. In the literature, it has been stated that decrease in tGSH, SOD, and CAT levels is due to the excessive expenditure of antioxidants while neutralizing ROS (Poljsak et al., 2013). Oxidative stress occurs when the production of ROS exceeds enzymatic and non-enzymatic antioxidant defenses (Burton and Jauniaux, 2011). Information acquired from the literature indicates that the oxidant/antioxidant balance in the heart tissue changes in favor of oxidants, and oxidative stress develops during Co exposure.

On the other hand, Co has been reported to induce the production of proinflammatory cytokines via a mechanism involving NF-κB signaling pathways (Mou et al., 2012). NF-κB has been shown to be a transcription factor activated in the presence of oxidative stress and is also useful as a biomarker for cellular oxidative stress (van den Berg et al., 2007). One of the main roles of NF-κB is to regulate the production of proinflammatory cytokines that contribute significantly to the inflammatory response (Zhang et al., 2024). TNF-α, IL-1β, and IL-6 are the most focused cytokines in the inflammatory response induced by Co (Yan et al., 2018). From our results, it was determined that rats treated with CoCl_2_ had significantly higher levels of NF-κB, TNF-α, IL-1β, and IL-6 in their heart tissues than rats of the control group. Oyagbemi et al. (2020) found that *albino Wistar-*type rats administered oral CoCL_2_ had higher levels of NF-κB expression in their heart tissues, which is consistent with the findings of this study. No information is available on the effect of Co on TNF-α, IL-1β, and IL-6 levels in the cardiac tissue. However, Abdel-Daim et al. (2020) reported that TNF-α and IL-6 levels increased in kidney tissues of rats exposed to CoCL_2_ for 4 weeks.

In this study, a significant increase was also observed in TpI and CK-MB levels in the serum of the CoCL_2_ group, which had high oxidant and proinflammatory cytokine levels in the cardiac tissue. Serum TpI and CK-MB levels are sensitive and reliable cardiac biomarkers for early detection of myocardial injury and necrosis (Roumeliotis et al., 2021). Liu et al. (2022) reported that CoCl_2_ increased ROS, MDA, TpI, and CK levels and decreased GSH, SOD, and CAT levels in cardiac myocytes. Our findings from the CoCL_2_ group are supported by evidence gathered from the literature.

In this study, it was observed that treatment with CE inhibited the increase in oxidant MDA levels and the decrease in antioxidant (tGSH, SOD, and CAT) levels in the heart tissue. Moreover, CE significantly suppressed the increase in proinflammatory cytokines (NF-κB, TNF-α, IL-1β, and IL-6) and cardiac markers (TpI and CK-MB). This result suggests that the effect of cinnamon is due to its antioxidant and free-radical scavenging properties (Ranasinghe et al., 2013), thereby reducing oxidative stress and NF-κB expression (Tuzcu et al., 2017) to decrease the production of proinflammatory cytokines and suppress inflammation (Ojcius et al., 2021).

As mentioned above, CoCL_2_ induces oxidative and inflammatory damage in heart and kidney tissues by decreasing antioxidant levels and increasing proinflammatory cytokine production (Oyagbemi et al., 2020). In the present study, CoCL_2_ increased oxidant and proinflammatory parameters in the kidney tissues of animals compared with the control group and decreased antioxidant parameters. These findings imply that Co treatment also affects the kidneys of animals. There are studies reporting that Co administration causes oxidative and inflammatory damage to the kidneys (Abdel-Daim et al., 2020; Oyagbemi et al., 2020; Iji et al., 2023). CE administration to animals suppressed the increase in oxidant and proinflammatory parameters and the decrease in antioxidant parameters. These findings are in accordance with those of Elshopakey and Elazab (2021), who reported that cinnamon treatment suppressed the increase in oxidant and proinflammatory levels and the decrease in antioxidant levels in the kidney. As is known, kidney damage leads to kidney dysfunction. Creatinine and BUN levels increase in renal failure, owing to acute kidney injury. Determination of serum creatinine and BUN levels is of great value in determining renal function (Lyman, 1986). The experimental results of this study show that CoCL_2_ administration increased serum BUN and creatinine levels. Treatment with CE significantly suppressed the Co-induced increase in creatinine and BUN levels. These findings are in agreement with the study of Tanomand et al., showing that cinnamon treatment decreased creatinine and BUN levels in *albino Wistar-*type rats (Tanomand and Najafian, 2013). Lusiana et al. (2022) observed that the active polyphenol in the form of cinnamaldehyde and cysteine in CE reduces urea activity by alleviating oxidative stress in the kidneys.

The liver is responsible for the detoxification of drugs and toxic chemicals. The present study revealed that CoCL_2_ increased oxidants and proinflammatory parameters and decreased antioxidants in the liver tissue of rats. These results allude that Co also has an impact on animal livers. According to Gonzales (2005), Co caused the liver of rats to produce more oxidants and less antioxidants. There is additional evidence that Co administration led to a large increase in proinflammatory cytokine indices in the liver (Abdel-Daim et al., 2020). Our experimental findings show that CE counteracted the impact of CoCL_2_ on oxidant, antioxidant, and proinflammatory parameters in the liver. Previous research has shown that the phenols and aldehydes in cinnamon have significant liver-protective functions (Ju, Santana de Oliveira and Qiao, 2023). In parallel with this study, Berktas and Peker showed that cinnamon treatment prevented the increase in MDA levels and the decrease in tGSH, SOD, and CAT levels in the rat liver (Berktas and Peker, 2021). Scavenging ROS has been shown to lead to the deactivation of NF-κB and the subsequent inhibition of proinflammatory cytokine release (Azab et al., 2011). In addition, Co increased ALT and AST activities in the serum of animals in this study. ALT and AST are enzymes that are mainly localized in liver cells and catalyze the transfer of amino groups between amino acids and keto acids (Xu et al., 2018). ALT is found in the cytosol of hepatocytes (Kaplan, 2002), whereas AST is found both in the cytosol and predominantly in the mitochondria (approximately 80%) of hepatocytes (Purbayanti and Nafarin, 2019). When hepatocytes are damaged, the permeability of their cell membrane increases, resulting in the release of ALT and AST from the liver cells into the bloodstream and causing their levels to increase in the serum (Xu et al., 2018). CE significantly suppressed the Co-induced increase in ALT and AST activities. This suggests that cinnamon may reduce Co-induced liver damage by stabilizing the hepatocyte membrane. Miah et al. (2022) also reported that cinnamon administered at different doses to *Swiss albino* mice decreased ALT and AST activities.

## 5 Conclusion

Co induced the production of oxidants and proinflammatory parameters and the consumption of antioxidants in the heart, kidney, and liver tissues of rats. Heart, kidney, and liver tissues were affected by Co damage with the same severity. The findings of this study provided an accurate characterization of the extent to which the heart, kidney, and liver are affected by Co toxicity. CE significantly prevented the increase in MDA, NF-κB, TNF-α, IL-1β, and IL-6 levels and the decrease in antioxidant levels in the heart, kidney, and liver of CoCL_2_-administered animals. A decrease in heart damage was detected by a decrease in TpI and CK-MB in blood serum, a decrease in kidney damage was detected by a decrease in creatinine and BUN levels, and a decrease in liver damage was also detected by a decrease in ALT and AST levels. Our experimental results indicate that CE protects heart, kidney, and liver tissues against oxidative and inflammatory damage caused by CoCL_2_. Thus, CE may be useful in the treatment of Co-induced oxidative and inflammatory heart, kidney, and liver damage. Further detailed studies are needed in the future to clarify the mechanisms of cardiac, renal, and hepatoprotective effects of cinnamon against Co toxicity.

## 6 Limitations

To further assess whether the heart, kidney, and liver are affected by Co toxicity and the restoring effect of CE, we recommend a histopathologic examination of each organ. Additionally, the lack of immunohistochemical detection of proinflammatory cytokine proteins in different organ tissues is among the limitations to the study.

## Data Availability

The original contributions presented in the study are included in the article/Supplementary Material; further inquiries can be directed to the corresponding author.
